# Annales D’Oculistique

**Published:** 1896-10

**Authors:** 


ANNALES D'OCULISTIQUE. Edited in Paris, by Dr. D. E. Sulzer and Dr. Valude for the French edition, and in New York by Dr. George T. Stevens for the English edition. The Transatlantic Publishing Company, 63 Fifth Avenue, New York. Subscription price of English edition is $5.00 a year.

This journal has been reviewed before in these pages. The April number, being No. 4 of Volume CXV, has been received. Among its most valuable articles is one entitled "Nasal Affections and Reflex Ocular Disturbances," by Laurens; "The Formation of Images in Regular Astigmatic Systems," by G. Weiss. This is, perhaps, the most important article in the book, and is finely illustrated. Another article is entitled "Large Subconjunctional Injections of Cyanide of Mercury in Infectious Keratitis," by Dr. Camille Fromaget. There are several shorter articles and some reports of societies, reviews of journals, and miscellaneous notes. Every well-educated physician should read this journal, containing, as it does, so much that is new and scientific upon the subject of that delicate and precious organ, the eye.

